# Advanced Deep Learning Approaches in Detection Technologies for Comprehensive Breast Cancer Assessment Based on WSIs: A Systematic Literature Review

**DOI:** 10.3390/diagnostics15091150

**Published:** 2025-04-30

**Authors:** Qiaoyi Xu, Afzan Adam, Azizi Abdullah, Nurkhairul Bariyah

**Affiliations:** 1 Center for Artificial Intelligence Technology, Faculty of Information Science and Technology, Universiti Kebangsaan Malaysia, Bangi 43600, Selangor, Malaysia; afzan@ukm.edu.my (A.A.); azizia@ukm.edu.my (A.A.); 2 College of Intelligent Manufacturing, Zibo Vocational Institute, Zibo 255314, China; 3 Department of Pathology, Faculty of Medicine, Universiti Kebangsaan Malaysia, Jalan Yaacob Latif, Bandar Tun Razak, Cheras 56000, Wilayah Persekutuan Kuala Lumpur, Malaysia; p110546@siswa.ukm.edu.my

**Keywords:** breast cancer, detection algorithm, whole slide images, deep learning, systematic literature review

## Abstract

**Background:** Breast cancer is one of the leading causes of death among women worldwide. Accurate early detection of lymphocytes and molecular biomarkers is essential for improving diagnostic precision and patient prognosis. Whole slide images (WSIs) are central to digital pathology workflows in breast cancer assessment. However, applying deep learning techniques to WSIs presents persistent challenges, including variability in image quality, limited availability of high-quality annotations, poor model interpretability, high computational demands, and suboptimal processing efficiency. **Methods:** This systematic review, guided by the Preferred Reporting Items for Systematic Reviews and Meta-Analyses (PRISMA), examines deep learning-based detection methods for breast cancer published between 2020 and 2024. The analysis includes 39 peer-reviewed studies and 20 widely used WSI datasets. **Results:** To enhance clinical relevance and guide model development, this study introduces a five-dimensional evaluation framework covering accuracy and performance, robustness and generalization, interpretability, computational efficiency, and annotation quality. The framework facilitates a balanced and clinically aligned assessment of both established methods and recent innovations. **Conclusions:** This review offers a comprehensive analysis and proposes a practical roadmap for addressing core challenges in WSI-based breast cancer detection. It fills a critical gap in the literature and provides actionable guidance for researchers, clinicians, and developers seeking to optimize and translate WSI-based technologies into clinical workflows for comprehensive breast cancer assessment.

## 1. Introduction

Breast cancer remains one of the most prevalent and deadly malignancies affecting women worldwide, posing a significant threat to both health outcomes and quality of life [1,2,3]. Despite remarkable advances in medical diagnostics and therapeutic strategies, the molecular heterogeneity and diverse clinical manifestations of breast cancer continue to complicate its detection and management [4]. Biomarker and lymphocyte detection are critical components in breast cancer assessment, offering essential guidance for diagnosis, classification, and prognosis [5].

Among these, the estrogen receptor (ER) serves as a key molecular marker for subtype classification and supports the development of personalized treatment regimens, directly influencing patient outcomes [6]. The proliferation index Ki-67 Antigen (Ki67) provides independent predictive value for treatment response, while the joint evaluation of ER and progesterone receptor (PR) further refines hormone therapy decisions [7]. Overexpression of human epidermal growth factor receptor 2 (HER2) correlates with increased tumor aggressiveness and recurrence risk, making HER2-positive cases suitable for targeted therapy. In addition, tumor-infiltrating lymphocytes (TILs) are considered reliable prognostic indicators, especially for triple-negative breast cancer (TNBC) patients, where TIL density correlates with both disease-free and overall survival [8]. Accurate identification of these biomarkers and immune features forms the foundation for effective clinical decision-making.

Recent advancements in whole-slide imaging (WSI) have enabled digital pathology to visualize entire tissue sections at ultra-high resolution, capturing both the spatial distribution of biomarkers and the microenvironmental context of lymphocytes [9,10]. These large-format digitized slides—typically prepared using Hematoxylin and Eosin (H&E) staining for structural visualization and Immunohistochemistry (IHC) for highlighting specific molecular targets—provide pathologists with richer information but also introduce significant technical challenges. The sheer volume of image data—often exceeding one billion pixels per slide—and the complex spatial patterns of diagnostic features render manual analysis time-consuming, subjective, and difficult to scale [11,12]. Traditional workflows depend heavily on expert interpretation, leading to potential variability and limited reproducibility in large-scale clinical studies.

To address these limitations, deep learning has emerged as a promising approach for automating WSI analysis. While segmentation tasks aim to label each pixel according to tissue type or structure [13], detection-based techniques are particularly well-suited for clinical applications. They generate interpretable, quantitative outputs such as cell counts, biomarker localization, and lymphocyte spatial distributions, which align more directly with diagnostic workflows [13,14]. This review focuses on detection-oriented methods in WSI analysis and their applications across four critical clinical tasks: diagnosis, classification, grading, and prognosis.

Nonetheless, the integration of deep learning into WSI-based breast cancer assessment remains nontrivial. The ultra-high resolution of WSIs demands substantial computational resources and presents design challenges for conventional model architectures [15,16]. Furthermore, WSIs exhibit a multi-scale nature—ranging from microscopic cell morphology to macroscopic tissue organization—necessitating models capable of learning across spatial hierarchies [17]. The analysis is further complicated by sparse diagnostic features, inter-sample heterogeneity, and staining variations. Compounding these issues is the scarcity of large, well-annotated datasets, which limits the scalability of supervised learning approaches [18]. Interpretability also remains a major concern, as the opaque decision-making processes of deep learning models reduce clinical trust [19]. Effective detection algorithms must therefore balance sensitivity to subtle morphological cues with robustness against noise and artifacts—especially when identifying key features such as biomarker expression and lymphocyte infiltration.

Despite a growing body of literature in this domain, existing reviews often remain narrowly focused. Many concentrate on segmentation techniques or algorithmic performance metrics, without sufficiently addressing the clinical integration of detection methods or their practical utility [20,21,22]. Additionally, critical issues such as dataset bias, computational burden, and feedback mechanisms from clinical deployment are frequently overlooked. This gap highlights the need for a more holistic and clinically grounded synthesis of detection-focused research in breast cancer WSIs.

To this end, this review aims to address three key research questions:

1. What types of datasets are used for comprehensive breast cancer assessment using WSIs?
2. What are the main challenges associated with comprehensive breast cancer assessment using WSIs?
3. How do WSIs impact the accuracy and reliability of advanced deep learning approaches for comprehensive breast cancer assessment?

**Figure 1:**
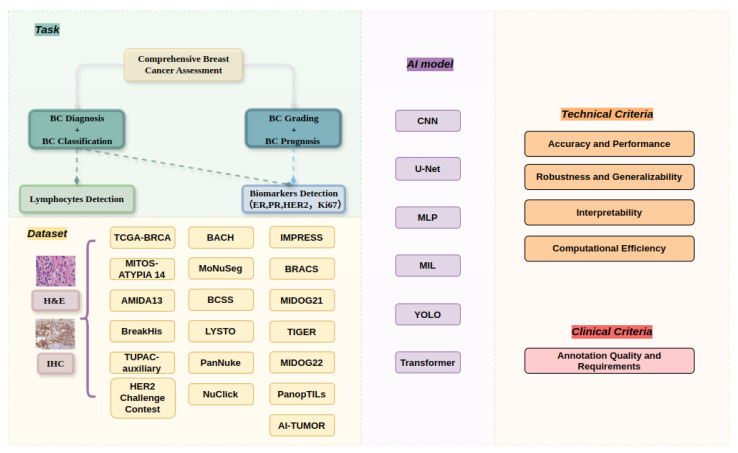
Overview of detection tasks, datasets, models, and evaluation criteria in WSI-based breast cancer assessment.

Figure 1 summarizes the task types, datasets, AI models, and evaluation criteria that frame this review of deep learning-based breast cancer assessment using WSIs. This study systematically reviews the landscape of relevant approaches published between 2020 and 2024, following PRISMA guidelines [23]. It synthesizes recent methodological advancements, identifies unresolved challenges, and outlines future research directions. In particular, we emphasize the clinical significance of detection tasks, the scalability of AI-based diagnostic tools, and the potential for deep learning to transform biomarker discovery and personalized treatment planning in digital pathology.

## 2. Methods

The study followed the PRISMA guideline [24] in this systematic literature review. PRISMA provides a consistent, repeatable process for locating, assessing, and selecting pertinent research. It also provides guidance on how to choose, recognize, and evaluate studies [25,26].

**Figure 2:**
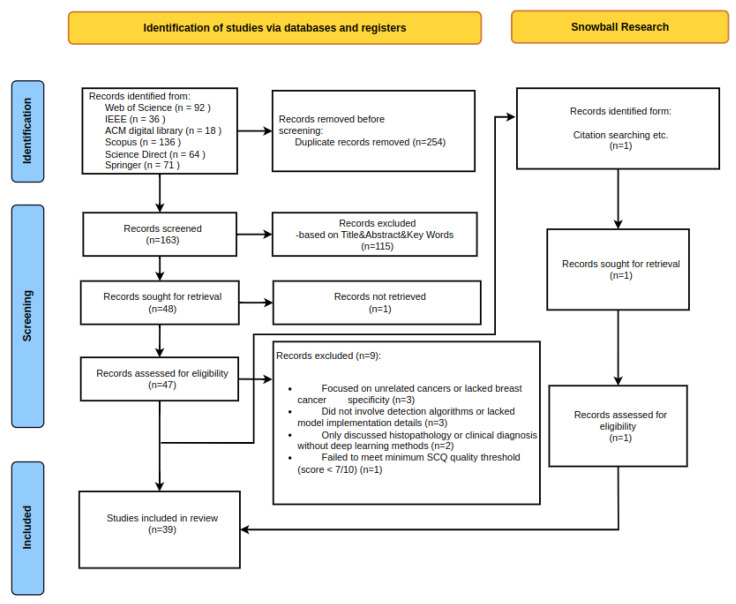
PRISMA flow diagram of the SLR.

Figure 2 shows the PRISMA procedure used for this systematic literature review. The next subsection provides details on the review process.

### 2.1. Data Sources and Search Strategy

To identify relevant academic publications, 8 major bibliographic databases were used, including Scopus, IEEE Xplore Library, Web of Science, SpringerLink, ACM Digital Library, and ScienceDirect. Consequently, the systematic literature review focused on articles published between 2020 and 2024 to ensure the inclusion of the most recent and relevant research findings. The search strategy employed combinations of key terms like the following:

- “breast cancer detection” AND “deep learning”
- “breast cancer diagnosis” AND “deep learning”
- “convolutional neural networks” AND “breast cancer”
- “lymphocytes detection” OR “biomarkers detection” AND “breast cancer”
- “H&E stained images” AND “deep learning” AND “breast cancer”
- “immunohistochemistry” AND “deep learning” AND “breast cancer”
- “automated breast cancer diagnosis” OR “AI in breast cancer screening”

These search parameters were created to encompass the depth of knowledge regarding deep learning applications in the detection and analysis of breast cancer.

### 2.2. Selection Criteria

An exhaustive search strategy combining both automated and manual methods initially retrieved 417 academic publications. Throughout the entire screening process, the inclusion and exclusion criteria outlined in Table 1 were applied consistently. After eliminating 254 duplicate records, 163 unique publications were screened based on their titles, abstracts, and keywords. At this stage, 115 papers were excluded due to a lack of domain relevance or methodological inadequacy.

Following this, 48 full-text articles were retrieved for in-depth evaluation. One article was excluded due to inaccessibility, and the remaining 47 underwent full eligibility assessment. Nine additional studies were excluded for the following reasons: three did not focus specifically on breast cancer or its primary clinical tasks (e.g., detection, classification, prognosis); three lacked concrete details regarding deep learning algorithms or implementation strategies; two relied solely on conventional pathology without computational approaches; and one failed to meet the minimum quality threshold on the Standard Quality Checklist (SCQ).

This systematic and criteria-driven selection process resulted in 39 high-quality studies being included in the final review. The characteristics of these selected publications are summarized in Table 2, providing a foundation for subsequent quality assessment and synthesis.

### 2.3. Quality Assessment

Assessing the quality of evidence is crucial in a systematic literature review (SLR), as methodological biases may influence outcomes and lead to misinterpretation. To ensure the reliability and rigor of the included studies, this review adopted the Standard SCQ proposed by [66], which comprises ten evaluation items. Following the approach of [57], only studies that provided a “yes” response to at least seven SCQ items were included.

Among the 39 studies selected after quality filtering, the SCQ score distribution was as follows: 10 studies received a full score (10/10), 15 studies scored 9/10, 11 studies scored 8/10, and 3 studies met the minimum threshold with 7/10. Studies scoring below this threshold were excluded to maintain methodological rigor. This distribution reflects the overall quality consistency of the selected literature and ensures that only robust and dependable studies were included in the final synthesis. The SCQ evaluation and data extraction processes were closely integrated to enhance the validity and significance of the review outcomes. Table 3 outlines the SCQ criteria used in this study.

### 2.4. Data Extraction and Synthesis

The study noted the pertinent information from each study, such as the publisher, authors, and year of publication, and gathered data for our SLR on the deep learning techniques used, reported accuracy, and assessment criteria. To answer the study’s research questions, the authors particularly examined the results during the data synthesis phase. The authors used a variety of visualization approaches and tools, including tables and diagrams, to make this analysis easier.

## 3. Results and Meta-Analysis

The meta-analysis of the search results from our systematic literature review is shown in this section. It starts with a summary of the chosen articles before going into each of the study questions that were developed and stated in the introduction.

### 3.1. Overview of Selected Studies

**Figure 3:**
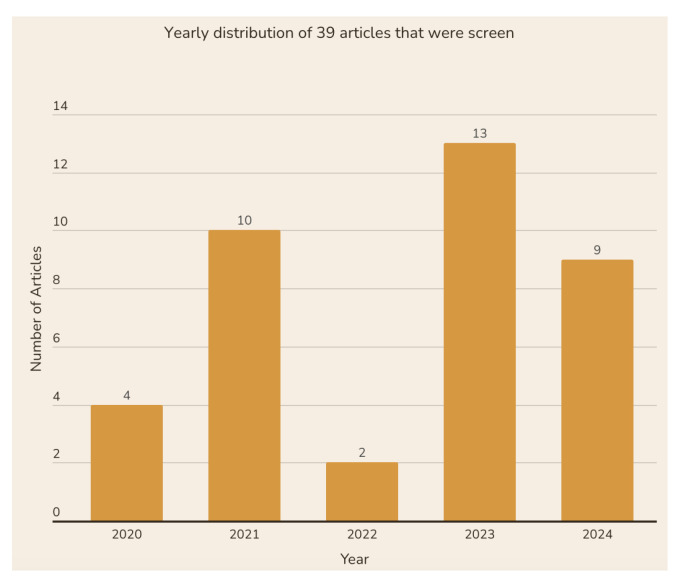
Diagram showing the number of articles published per year from 2020 to 2024.

Figure 3 provides a chronological overview of the articles selected for this SLR, detailing the number of publications related to advanced deep learning methods for breast cancer cell detection using digital pathology images from 2020 to 2024. The diagram indicates a growing trend in this research area over the past few years, particularly from 2023 to 2024, when the number of published articles significantly increased. Most of the articles considered for this study were published after 2023. Specifically, the highest number of articles published in a single year was 13 in 2023 and 9 in 2024, followed by 10 papers in 2021 and 4 papers in 2020. In contrast, only 2 papers were reviewed in 2022, which some literature attributes to the impact of the COVID-19 pandemic on research output.

### 3.2. Research Question 1: What Types of Datasets Are Employed for Comprehensive Breast Cancer Assessment Using WSIs?

This research evaluates 20 key WSIs datasets pivotal to advancing deep learning applications in breast cancer detection, diagnosis, classification, grading, and prognosis. These datasets, each contributing uniquely to comprehensive breast cancer assessment, reflect the rapid evolution and diversification of digital pathology resources.

The following attributes define the state of the breast cancer WSI datasets:

1. Scale and Origin:
  - The basic resources are large-scale public repositories such as TCGA-BRCA (3111 WSIs) [67].
  - Focused, high-resolution data are available from specialized datasets like PanNuke (200,000 nuclei across 19 tissue types) and BACH (400 patches, 30 WSIs) [68,69].
  - For particular study goals, derivative datasets (MoNuSeg, BCSS, TIGER) expand on primary sources [70].
2. Research Focus and Annotation Granularity:
  - Datasets cover a wide range of anatomical structures, such as nuclei in MoNuSeg and tumor-infiltrating lymphocytes in PanopTILs, to specific cellular structures.
  - Increasing model sophistication is reflected in the variation in annotation detail from whole-slide to pixel-level [71].
3. Multi-modal Integration:
  - Datasets that integrate clinical and genetic information with histopathological images, such as TCGA-BRCA, provide opportunities for more thorough and comprehensive analysis [72,73].
4. Ethical Considerations and Diversity:
  - More recent datasets, such as AI-TUMOR, highlight the diversity of patient demographics and the use of ethical data collection techniques [74].

Together, these datasets enable a wide range of deep learning uses, ranging from simple cell identification to intricate tumor categorization and prognostic modeling. In addition to driving innovation in deep learning architectures suited to the particular difficulties of breast cancer analysis, they act as benchmarks for model development and validation [75,76].

These datasets development follows the field’s move toward more extensive, fully annotated resources on a wider scale. This pattern suggests a developing sector ready to use cutting-edge computational techniques to address the intricate, multidimensional character of breast cancer research, along with the integration of multimodal data and a growing emphasis on ethical issues.

Table 4 lists the various datasets and highlights their unique properties that were employed for WSI-based comprehensive breast cancer assessment. Each dataset plays a crucial role in advancing deep learning methodologies by providing diverse and detailed data for model training and validation.

### 3.3. Research Question 2: What Are the Main Challenges Associated with Comprehensive Breast Cancer Assessment Using WSIs?

WSI has emerged as a pivotal component in the realm of digital pathology, particularly for the application of deep learning methodologies in the detection of breast cancer [90]. Nonetheless, numerous substantial obstacles hinder its optimal implementation. These obstacles encompass a spectrum of technical challenges associated with image processing and analytical procedures, as well as overarching issues pertaining to data integrity, and annotation practices. Table 5 summarizes the main issues raised in recent research papers, together with their descriptions, implications for breast cancer detection, and relevant research references.

### 3.4. Research Question 3: How Do WSIs Affect the Accuracy and Reliability of Advanced Deep Learning Approaches for Comprehensive Breast Cancer Assessment?

WSI plays a critical role in enhancing the accuracy and reliability of deep learning techniques for comprehensive assessments of breast cancer [91]. Deep learning algorithms can examine complicated tissue structures and cellular configurations with more ease thanks to the high-resolution and finely detailed features of WSIs. This is crucial for distinguishing between cancerous and benign cells. However, as Table 6 summarizes, several critical variables related to WSIs significantly impact the accuracy and reliability of deep learning models when it comes to breast cancer diagnosis.

As Table 6 shows, these factors are interrelated and impact model performance. Reliability in various circumstances is contingent upon constant image quality and standardization, whereas high resolution improves detection accuracy but raises processing demands. Strong annotations and a wide range of training data are essential for the resilience and generalizability of the model. WSI resolution makes it easier to obtain the necessary detail for accurate analytical evaluations, but it also creates problems with data processing and standardization methods. It is crucial to guarantee that WSIs are of superior quality, coherent, and comprehensively annotated, while concurrently tackling the intricacies linked to data heterogeneity and standardization since it is essential to the creation of reliable models that may be successfully applied in clinical settings.

In summary, WSIs have a major influence on the precision and dependability of deep learning techniques used in the detection and assessment of breast cancer. Developing deep learning models that are therapeutically useful requires addressing these variables simultaneously. In order to further improve the accuracy and dependability of deep learning techniques in breast cancer evaluation utilizing WSIs, future research should concentrate on maximizing these factors.

## 4. Criteria for Comprehensive Breast Cancer-Assessment WSI Algorithms

A number of important factors must be considered when evaluating detection criteria for a thorough assessment of breast cancer based on WSI algorithms to guarantee efficacy, consistency, and clinical applicability. These criteria can be divided into two factors: technological and clinical. Each of these factors is vital in establishing the technology’s overall value and suitability.

Technical Criteria:

1. Accuracy and Performance Metrics: According to [91], sensitivity, specificity, accuracy, recall, AUC, and F1 score are essential for accurately identifying cancer cells while reducing false positives.
2. Robustness and Generalizability: Algorithms must manage common problems like noise and artifacts while operating consistently over a range of datasets, scanners, and staining processes [92].
3. Interpretability and Explainability: Clinical trust depends on model openness and error analysis capabilities [93].
4. Computational Efficiency: Algorithms should be appropriate for a range of computational contexts, with respectable processing speeds and efficient resource consumption [94].

Clinical Criteria:

Annotation Quality and Requirements: Preference for algorithms that reduce reliance on resource-intensive annotations by performing well with minimum or semi-supervised learning [94].

Together, these criteria ensure that the algorithms chosen are not only sound from a methodological standpoint but also practical, significant, and useful in clinical settings. Through a methodical approach to these components, scientists and medical professionals can evaluate and choose algorithms that are suitable for the complex problems involved in WSIs-based breast cancer cell detection, improving diagnostic accuracy and patient outcomes. To drive progress in breast cancer-detection techniques, researchers must prioritize optimizing baseline models while also carrying out thorough assessments of those models. These models serve as essential baselines to assess the effectiveness of WSI-based detection techniques in clinical settings. Targeted optimization techniques can significantly enhance important characteristics like clinical relevance, accuracy, and robustness, addressing the many problems that come with assessing breast cancer. This systematic approach guarantees that the chosen algorithms enhance patient outcomes and boost diagnostic accuracy.

### 4.1. Baseline Models for Detection Technologies Applied in Comprehensive Breast Cancer Assessment Based on WSI Algorithms

**Figure 4:**
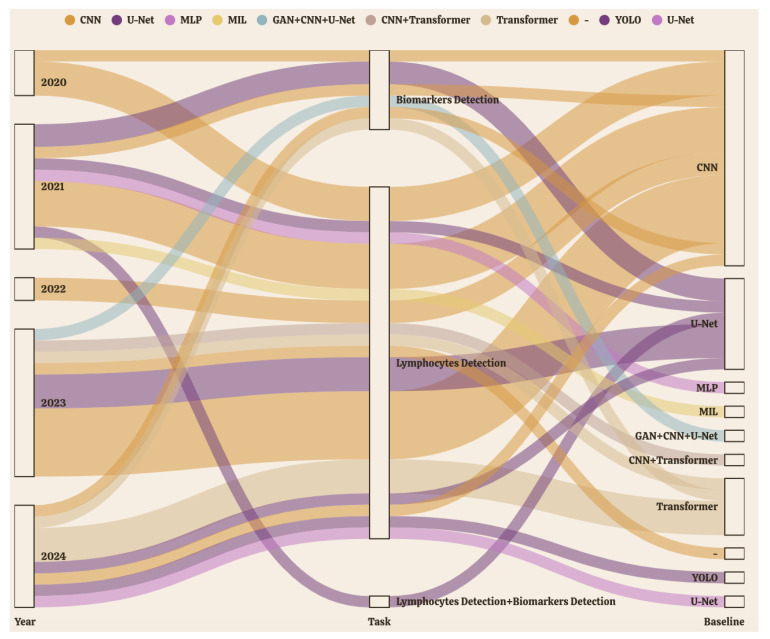
Evolution of baseline-detection models for breast cancer assessment in WSI from 2020 to 2024. (Flow width indicates the relative usage frequency of each model type, with thicker streams showing more widely adopted models. Different colors represent distinct model architectures tracked across the study period).

To better visualize the methodological progression in WSI-based breast cancer detection over recent years, Figure 4 presents a Sankey diagram capturing the dynamic interplay between publication year, detection task, and model architecture from 2020 to 2024. Each stream represents a flow of research attention, where the thickness of the connection reflects the frequency of model usage for specific tasks. As shown in the figure, two major trends emerge: first, a shift in detection focus—from early emphasis on biomarkers to increasing attention on lymphocyte detection, and more recently, to frameworks addressing both targets simultaneously; and second, a transition in model design—from dominant use of Convolutional Neural Network (CNN) and U-Net toward more sophisticated or hybrid approaches involving Transformers and Generative Adversarial Network (GAN), reflecting growing demands for richer spatial modeling and generalization across WSI domains.

These evolving trends are closely reflected in the selection and adaptation of baseline model architectures for different detection tasks. Over the five-year span, CNN and U-Net remained dominant. CNNs were predominantly applied to biomarkers detection, where classification or sparse detection was needed to identify ER/PR/HER2-positive cells. In contrast, U-Net architectures were widely adopted for lymphocyte detection, due to their pixel-level precision and strong segmentation capabilities—critical for detecting densely distributed immune cells with indistinct boundaries. Notably, in tasks combining biomarkers and lymphocyte detection, U-Net-based frameworks were still preferred for their ability to support multi-task outputs, such as simultaneous localization and segmentation.

From 2022 onward, architectural diversification accelerated. Transformer-based models gained traction, especially in biomarker detection, by leveraging self-attention mechanisms to capture long-range contextual dependencies in high-resolution WSI data. Hybrid approaches, such as CNN+Transformer and GAN+CNN+U-Net, emerged around 2023, integrating the spatial locality of CNNs, the generative robustness of GANs, and the global modeling power of Transformers—enabling more adaptive and domain-generalizable detection systems. Meanwhile, lighter or exploratory models like Multilayer Perceptron (MLP), Multiple Instance Learning (MIL), and You Only Look Once (YOLO) appeared after 2021, though their use remained limited due to challenges in dense detection and precise localization on WSIs.

Evaluation metrics across these models varied with task and output granularity. Classification and sparse detection tasks typically employed AUC, accuracy, and F1-score, while segmentation-oriented models were assessed using Dice coefficient, IoU, and boundary-aware metrics. For multi-output detection models, task-specific metrics were reported independently, reflecting the complexity of comprehensive breast cancer assessment.

Overall, segmentation-centric models have remained the backbone of WSI-based detection. Their ability to handle high-resolution images through patch-based processing, preserve pixel-level detail, and support dense prediction tasks makes them especially suited for WSI applications, where targets are often overlapping, small, and structurally complex. The increasing use of hybrid and Transformer-based models reflects a broader trend toward unifying global and local representation learning for improved clinical utility.

### 4.2. Optimizing and Improving Existing Baselines Based on Evaluation Criteria

A multidisciplinary approach is required to optimize current baseline models for breast cancer cell detection using WSI, considering technological and clinical factors. Several important categories can be used to group this optimization process:

#### 4.2.1. Enhancing Model Performance

In the realm of deep learning-based detection techniques for computer-aided diagnosis (CAD) of breast cancer, strategies to enhance model performance have become increasingly diverse and sophisticated. Primarily, ensemble learning frameworks effectively improve detection accuracy and generalization by integrating various deep learning architectures such as U-Net, GANs, and CNNs [36,42,43,44,45,46,48,49,50,51,53,54,55,58,59,62,65]. Additionally, the application of multi-task learning paradigms, which allow models to jointly learn multiple related tasks such as lesion segmentation, classification, and malignancy grading within a unified architecture, has gained attention for its ability to leverage shared representations, reduce overfitting, and improve generalization across heterogeneous lesions. This approach not only provides more comprehensive pathological information but also enhances the model’s robustness and diagnostic performance [43,53,56,59,61,63]. Furthermore, multimodal data fusion strategies integrate genomic data with WSIs [27,41], or combine multi-level data from cellular and tissue levels [37,57,60], significantly boosting diagnostic precision and interpretability by capturing complementary biological features. Training on cross-institutional, multi-center datasets enhances the model’s domain adaptability, mitigating the adverse effects of data distribution shifts on performance [34].

Regarding model architectures, increasing network depth equips models with stronger feature extraction capabilities, enabling the capture of more complex pathomorphological features [30]. The introduction of diverse convolutional modules, such as residual convolutional blocks [38], parallel convolutional blocks [35], dilated convolutional blocks [51], and color deconvolution [50], further enhances the model’s feature representation ability and multi-scale information capture. Recently, the integration of multiple attention mechanisms, including combinations of spatial attention, channel attention, and self-attention, has gained widespread application in medical image analysis, improving detection precision and efficiency by enhancing the model’s focus on key regions and features.

Lastly, post-processing optimization techniques, including morphological opening operations, watershed algorithms [32,38], and advanced methods like HoVer-Net [63], further elevate the precision and consistency of detection outcomes, particularly in cell instance segmentation and separation of adhered structures. The synergistic effect of these methods is driving the gradual implementation and application of breast cancer CAD systems in clinical practice.

While recent advances have significantly improved detection accuracy, they often come at the expense of interpretability and computational efficiency. In deep learning-based CAD systems, increasing model complexity to boost performance tends to reduce transparency and raises deployment barriers. For example, models like CB-HVT Net, which integrate PVT, ResNet variants, and attention mechanisms, feature high parameter counts and computational demands that hinder real-time deployment [45,54]. These challenges highlight the need for balanced approaches that maintain strong accuracy while enhancing explainability and deployability.

#### 4.2.2. Improving Robustness and Generalizability

In the realm of deep learning-based breast cancer-detection research, robustness enhancement strategies predominantly converge on two pivotal methodologies. Cross-disease data integration and validation, referring to the inclusion of datasets from multiple cancer types to expose the model to a broader range of pathological variations, substantially augments model robustness through the incorporation of multi-disease datasets and the execution of pan-cancer experiments [34,37,46,48,50,54]. This approach facilitates the model’s acquisition of heterogeneous pathological features, thereby enhancing its discriminative capacity across diverse cancer types and elevating its adaptability within complex clinical milieus. Concurrently, the implementation of semi-supervised learning paradigms efficaciously addresses the constraints imposed by the paucity of annotated data [29]. By synergistically leveraging limited labeled datasets in conjunction with voluminous unlabeled data, this technique not only mitigates the reliance on extensive manual annotation processes but also markedly amplifies the model’s generalization prowess in data-constrained environments.

However, while cross-disease integration can enhance the model’s adaptability to diverse histopathological morphologies, it may also introduce label noise and inconsistencies in annotation standards. This, in turn, can lead to domain shift and reduce detection specificity for certain cancer types, such as breast cancer. For example, when independent models were constructed for five cancer types in TCGA and pan-cancer training was applied, the performance of pan-cancer models declined in certain tasks (e.g., PD-L1 prediction in STAD), suggesting that disease-specific signals may be diluted in a multi-cancer setting [37]. Moreover, many semi-supervised frameworks rely on heuristic thresholds or consistency regularization strategies, which often require task-specific tuning, thereby limiting their generalizability and scalability in real-world clinical applications.

#### 4.2.3. Increasing Interpretability and Explainability

Interpretability techniques in deep learning have emerged as essential components for fostering trust, transparency, and clinical acceptability in breast cancer assessment. The Human-Interpretable Features (HIF) paradigm aims to bridge the semantic gap between model outputs and clinical understanding by aligning predictions with visually and diagnostically meaningful image features [37], whereas saliency-based visualization methods offer intuitive heatmaps that localize regions contributing most to the model’s decision-making process, thereby enhancing interpretability for end-users [40]. While the integration of such methods has demonstrably improved the transparency and perceived reliability of AI-assisted diagnostic systems, several limitations persist. HIF-based strategies typically rely on predefined, handcrafted feature sets, which may insufficiently capture the complex and abstract representations encoded by deep neural networks, thus constraining their explanatory power and generalizability across datasets or imaging modalities. In parallel, saliency visualizations are often susceptible to input perturbations and architectural variations, producing unstable and sometimes misleading attributions. Moreover, the post hoc nature of most interpretability tools, coupled with a lack of standardized validation protocols, raises concerns regarding their clinical robustness and reproducibility. These limitations underscore an urgent need for the development of principled, rigorously evaluated interpretability frameworks that can yield consistent, meaningful, and clinically actionable explanations.

#### 4.2.4. Optimizing Computational Efficiency

In research applying deep learning techniques to breast cancer assessment, various strategies have been employed to enhance model efficiency. The primary approach involves precise localization of ROI through methods such as Gaussian kernel annotation [32] and micro-block selection techniques [31,64], enabling models to focus on key pathological features. This not only improves accuracy but also reduces computational costs. Additionally, researchers have utilized pre-training strategies to optimize model architecture [29,31], accelerating model convergence, improving task initialization, and reducing parameter count and architectural complexity, thereby lowering computational demands while maintaining high performance. However, these efficiency-oriented strategies are not without limitations. ROI localization methods often rely on heuristic rules or expert-defined annotations, which may introduce bias and reduce scalability across datasets with varying staining characteristics or image resolutions. Moreover, such narrowly focused techniques risk omitting relevant contextual cues essential for accurate diagnosis. Similarly, the effectiveness of pre-training heavily depends on the relevance and quality of the source domain; mismatched pre-training can result in suboptimal initialization and diminished downstream performance. Furthermore, existing studies rarely provide systematic evaluations of the trade-offs between architectural simplification and diagnostic accuracy, leaving the optimal balance between efficiency and performance largely unexplored.

#### 4.2.5. Addressing Data Quality and Annotation Challenges

In deep learning-based breast cancer-detection research, data quality and annotation challenges are primarily mitigated through two methodologies: weak supervision learning and segmentation map generation. Weak supervision enables models to extract features from limited or imprecise annotations, reducing dependence on fully annotated datasets [29,31,41]. Segmentation map generation techniques create synthetic annotations, providing finer-grained information on regions of interest, thus compensating for incomplete or noisy labels [39]. These approaches synergistically enhance model robustness and performance in the face of data quality and annotation challenges in breast cancer-detection tasks. However, both methods present notable limitations. Weakly supervised models are vulnerable to label noise and may overfit to coarse annotations, limiting their generalizability. Meanwhile, segmentation map generation often relies on heuristic or rule-based pseudo-labels that may introduce bias, particularly in complex tumor microenvironments. The lack of standardized validation procedures for these synthetic annotations also raises concerns regarding their clinical reliability and reproducibility.

Table 7 concisely summarizes the baseline models and their optimization strategies, aiding in the understanding of how these models can be improved to enhance the efficiency of comprehensive breast cancer assessment based on detection technologies.

## 5. Discussion and Potential Solutions for Improving WSIs for Breast Cancer Cell Detection

The application of advanced deep learning methodologies to WSIs for comprehensive breast cancer assessment represents a significant advancement in digital pathology. A systematic analysis of WSI datasets, associated challenges, and their impact on deep learning models has yielded five critical evaluation metrics: model performance, data integration and preprocessing, architectural optimization, robustness and generalizability, and interpretability and clinical application. This analytical framework provides a comprehensive evaluation of current technologies while illuminating future research trajectories.

The diversity of WSIs datasets, ranging from large-scale public repositories such as TCGA-BRCA to specialized high-resolution datasets like PanNuke and BACH, significantly influences model performance and data integration strategies. The multimodal nature of datasets such as TCGA-BRCA offers a robust foundation for developing comprehensive assessment models. However, dataset heterogeneity presents substantial challenges, particularly in terms of model generalizability. Innovative approaches have emerged to address these challenges. Notable among these is the integration of U-Net, GANs, and CNNs [43], which demonstrates exceptional performance in handling diverse data. Additionally, the multi-task learning paradigm described in [53] enhances model adaptability to varied WSIs data types through simultaneous segmentation, classification, and grading tasks.

The primary challenges in WSIs applications—including image size, quality variability, and annotation difficulties—directly impact data preprocessing strategies and model architecture design. These challenges have catalyzed innovative solutions. The panoramic segmentation method proposed by Liu et al. effectively addresses large-sized WSIs processing, markedly improving computational efficiency. In data integration and preprocessing, [27] showcases a multimodal fusion strategy combining genomic data with WSIs, enhancing diagnostic accuracy while partially mitigating data heterogeneity issues. Nevertheless, the efficient processing of large-scale WSIs data remains a significant challenge, necessitating further research into advanced preprocessing techniques.

The impact of WSIs on deep learning model accuracy and reliability reveals a dichotomy: high-resolution data enhances detection precision while simultaneously presenting computational and standardization challenges. This contradiction has driven innovations in model architectures. The introduction of residual convolutional blocks [38] significantly enhances feature extraction capabilities, while the attention mechanism described in [63] improves the model’s capacity to identify key regions. These optimizations directly address the computational challenges posed by high-resolution WSIs while enhancing the model’s ability to capture complex pathological features.

Regarding robustness and generalizability, cross-disease dataset integration [34] and semi-supervised learning methods [29] demonstrate significant advantages in managing diverse WSIs datasets and limited annotated data. These approaches enhance model adaptability across varied clinical settings and effectively address the challenge of WSIs data annotation difficulties.

Interpretability and clinical application remain significant hurdles for WSI-based deep learning models. HIF methods [37] and saliency visualization techniques [40] have advanced the interpretability of model outputs, crucial for fostering clinical trust and adoption. However, the seamless integration of these technologies into clinical workflows requires further investigation. The identified evaluation metrics not only provide a comprehensive assessment of existing models but also delineate key directions for future research. Significant opportunities persist in addressing WSIs dataset diversity, processing large-scale high-resolution data, and improving model robustness and interpretability.

## 6. Conclusions, Implication, and Recommendations for Future Research

This review has systematically examined deep learning-based detection methods for breast cancer using WSIs, highlighting substantial progress in model accuracy, robustness, and data efficiency. Nonetheless, significant challenges remain in computational scalability, interpretability, annotation quality, and clinical applicability. To address these issues, future research should be guided by both technical feasibility and clinical relevance. In the short term, efforts should focus on developing lightweight, interpretable architectures optimized for WSI-scale processing to support real-time, resource-aware deployment. Enhancing weakly supervised and semi-supervised learning frameworks through uncertainty modeling and confidence-guided label refinement represents a technically viable strategy for improving annotation robustness. Medium-term priorities include designing domain-adaptive and resolution-consistent models to address data heterogeneity across institutions and staining variations. In the long term, the establishment of clinically validated interpretability protocols and the construction of large-scale, standardized WSI datasets should be pursued to support reproducibility, benchmarking, and translational impact. Prioritizing these directions will facilitate a more effective alignment between algorithmic innovation and real-world clinical integration, advancing the role of AI in precision breast cancer diagnostics.

## Figures and Tables

**Table 1:** Inclusion and exclusion criteria.

Inclusion Criteria	Exclusion Criteria
Articles related to advanced deep learning-based techniques for comprehensive breast cancer assessment, focusing on diagnosis, classification, grading, and prognosis	Duplicates
Emphasis on peer-reviewed journal articles and conference papers	Articles not addressing breast cancer detection, classification, grading, or prognosis strategies
Studies employing WSI techniques and related fields, particularly those focusing on breast cancer diagnosis and prognosis	Studies not related to WSI techniques and their application in breast cancer assessment
Articles that meet rigorous quality-assessment standards and have a clear focus on the two major tasks of breast cancer assessment	Articles not meeting quality-assessment standards
Articles addressing breast cancer-detection techniques through WSIs, focusing on key points like biomarkers and lymphocytes	Studies not utilizing WSIs or not focusing on the specified key points for breast cancer assessment

**Table 2:** Summary of included studies with evaluation and data scale (LD = lymphocyte detection; BD = biomarker detection; - = not specified in the article).

Source	Year	Task	Model	Result	Dataset and Scale
1 [27]	2020	BD	AlexNet	F1 score = 0.73	Private Dataset 50 Ki67 WSIs 50 H&E WSIs
2 [28]	2020	LD	NucTraL + BCF	Accuracy = 0.9691	BreakHis Benign: 637 WSIs Malignant: 1390 WSIs
3 [29]	2020	LD	Deep-CNN + FSRM	Accuracy = 98.8%, F1 score = 0.967	BreakHis Benign: 2480 WSIs Malignant: 5429 WSIs
4 [30]	2020	LD	Faster R-CNN	F1 score: Scattered lymphocytes: 0.9615 Agglomerated lymphocytes: 0.8645 Artifact area: 0.8197	LYSTO 1228 WSIs
5 [31]	2021	BD	U-Net-piNET	F1 score: Ki67-: 0.868; Ki67+: 0.804	Private Dataset 1142 WSIs
6 [32]	2021	BD	U-Net	Pearson correlation coefficient of r = 0.95	Private Dataset 200 WSIs
7 [33]	2021	BD	DLS	AUC = 0.60	TCGA-BRCA 3274 WSIs
8 [34]	2021	LD	UBCNN	-	MoNuSeg 120 WSIs
9 [35]	2021	LD	Micro-Net + MLP	F1 score = 0.87	TCGA-BRCA 4281 nuclei images
10 [36]	2021	LD	UNMaSk	F1 score: H&E: 0.856 IHC: 0.9037	Private Dataset 178 WSIs
11 [37]	2021	LD	CNN	AUROC = 0.864	TCGA-BRCA 7075WSIs
12 [38]	2021	LD & BD	PathoNet	F1 score = 0.7928	SHIDC-BC-Ki-67 2357 WSIs
13 [39]	2021	LD	SegNet + U-Net	F1 score = 0.91	BCa 100 ROIs
14 [40]	2021	LD	WeakSTIL	-	TCGA-BRCA 286 WSIs
15 [41]	2021	LD	CNN	-	TCGA-BRCA 2358 WSIs
16 [42]	2022	LD	TDC-LC	F1 score = 0.892	LYSTO 19663 patches
17 [43]	2022	LD	DDTNet	F1 score = 0.907	TCGA-BRCA 865 WSIs
18 [44]	2023	BD	GAN+CNN+U-Net	-	Private Dataset 321 samples
19 [45]	2023	LD	CB-HVT Net	F1 score = 0.88	LYSTO & NuClick 21312 images
20 [46]	2023	LD	AC-Former	F1 score = 0.796	TCGA-BRCA 452 images
21 [47]	2023	LD	-	-	LYSTO 83 WSIs
22 [48]	2023	LD	CBAM- Residual U-Net	F1 score = 0.9	TNBC 81 WSIs
23 [49]	2023	LD	U-Net +Mask R-CNN	F1 score = 0.941	Private Dataset 63 WSIs
24 [50]	2023	LD	MobileNetV2 +U-Net	F1 score = 0.927	Private Dataset 30 WSIs
25 [51]	2023	LD	DC-Lym-AF	F1 score = 0.84	LYSTO & NuClick 871 images
26 [52]	2023	LD	FMDet	F1 score = 0.773	MIDOG21 MITOS-ATYPIA 14, AMIDA13, TUPAC-auxiliary 403 WSIs
27 [53]	2023	LD	DeepLabV3	F1 score = 71.23	OCELOT, TIGER 17041patches
28 [54]	2023	LD	BCF-Lym-Detector	F1 score: LYSTO: 0.93 NuClick: 0.84	LYSTO & NuClick 16326 images
29 [55]	2023	LD	CNN	F1 score: Immune cell: 0.82 Tumour cell: 0.92 Stromal cell: 0.81	Private Dataset 2549 images
30 [56]	2023	LD	PROACTING	AUC = 0.88	IMPRESS 1053 images
31 [57]	2024	BD	Based on CNN	AUC = 0.72	TCGA-BRCA 14435 images
32 [58]	2024	BD	ViT	F1 score = 0.9269	Her 2 challenge contest 172 WSIs
33 [59]	2024	LD	Cell-Tissue- ViT	F1 score = 0.7243	OCELOT 400 Patches
34 [60]	2024	LD	Tissue Context + Cell Refinement	F1-score = 0.7473	OCELOT 400 Patches
35 [61]	2024	LD	PathoNet	F1 score: SHIDC-B-Ki-67: 0.842 LSOC-Ki-67: 0.909	SHIDC-BC-Ki-67, LSOC-Ki-67 2447 WSIs
36 [62]	2024	LD	DeepMitosisNet	F1 score = 0.93	MITOS-ATYPIA 14 2127 images
37 [63]	2024	LD	CellViT	F1 score = 0.81	PanNuke 7959 images
38 [64]	2024	LD	YOLOv5	F1 score = 0.7438	TIGER 1879 ROIs
39 [65]	2024	LD	MuTILs	AUROC = 93.0	PanopTILs 1317 ROIs

**Table 3:** Checklist for quality assessment.

Checklist for Quality Assessment	SCQ No
Is the report coherent and easy to read?	SCQ1
Is the research’s purpose well-defined?	SCQ2
Is the procedure for gathering data clearly laid out?	SCQ3
Have the settings of diversity been thoroughly examined?	SCQ4
Can the study’s conclusions be trusted?	SCQ5
Is there a connection between the information, analysis, and conclusion?	SCQ6
Is the process of experimentation and approach transparent?	SCQ7
Are the research methods sufficiently documented?	SCQ8
If they are credible, are they important?	SCQ9
Is it possible to duplicate the research findings?	SCQ10

**Table 4:** Key WSI datasets relevant to comprehensive breast cancer assessment.

Name	Year	Key Features	Contribution	Link
TCGA-BRCA [77]	-	3111 H&E-stained WSIs from 1086 female and 12 male breast cancer patients. Includes matched gene expression data and clinical information.	Widely used to create derivative datasets like MoNuSeg, BCSS, LYSTO, and TIGER.	https://portal.gdc.cancer.gov/projects/TCGA-BRCA (accessed on 18 July 2024)
MITOS-ATYPIA 14	2013	2400 high-power field (HPF) images from 11 breast cancer patients, scanned by two devices: 1200 at 1539 × 1376 pixels and 1200 at 1663 × 1485 pixels, all at 40× magnification.	Provides a comprehensive external test set for evaluating the robustness of mitosis-detection models across different imaging conditions.	https://mitos-atypia-14.grand-challenge.org/Description/ (accessed on 18 July 2024)
AMIDA13 [78]	2013	606 HPF images (311 training, 295 test) from 23 subjects, each 2000 × 2000 pixels (0.25 mm^2^)	Established a benchmark for mitosis-detection algorithms, later integrated into larger datasets for broader impact.	-
BreakHis [79] (accessed on 18 July 2024)	2016	9109 breast tissue images from 82 patients at various magnifications (40×–400×). Includes 2480 benign and 5429 malignant samples, all 700 × 460 pixel RGB images. Samples collected via partial mastectomy.	Provides a diverse, well-annotated dataset for developing and evaluating breast cancer classification algorithms. Enables research on automated diagnosis across different magnifications and tumor types, potentially improving clinical diagnostic accuracy.	https://web.inf.ufpr.br/vri/databases/breast-cancer-histopathological-database-breakhis/ (accessed on 18 July 2024)
TUPAC-auxiliary [78]	2016	73 breast cancer WSIs from 3 pathology centers, scanned by 2 types of scanners at 40× magnification.	Integrates multi-center data, enhancing the scope of mitosis-detection research in breast cancer.	https://tupac.grand-challenge.org/ (accessed on 18 July 2024)
HER2 Challenge Contest [80]	2016	High-resolution breast cancer histology dataset. Comprises 100 gigapixel WSIs. Annotated with expert pathologist HER2 scores and percentage assessments.	Pioneering benchmark for automated HER2 quantification in digital pathology. Facilitates the development of AI tools to enhance diagnostic consistency and efficiency in breast cancer assessment.	https://warwick.ac.uk/fac/cross_fac/tia/data/her2contest/ (accessed on 18 July 2024)
BACH [81]	2018	Includes 400 H&E-stained patches (2048 × 1536 resolution) and 30 WSIs with pixel-level annotations	Useful for training models with pixel-level cancer type annotations	https://www.kaggle.com/datasets/truthisneverlinear/bach-breast-cancer-histology-images (accessed on 18 July 2024)
MoNuSeg [82]	2018	WSIs from 30 organs, creating 1000 × 1000 pixel sub-images with nuclear annotations.	Ensures variation in nuclear appearance, enhancing model training on diverse tissue samples.	https://monuseg.grand-challenge.org/Data/ (accessed on 18 July 2024)
BCSS [83]	2019	151 WSIs with representative regions of interest (ROI) selected, contributing to the TIGER dataset.	Helps in understanding tumor-infiltrating lymphocytes in HER2+ and triple-negative breast cancers.	https://bcsegmentation.grand-challenge.org/ (accessed on 18 July 2024)
LYSTO [47]	2019	LYSTO data set comprises 20,000 images from 43 patients with breast, colon, and prostate cancers. It’s structured with a patient-level split: 19 for training, 9 for validation, and 6 for testing, ensuring diverse representation across cancer types and stages.	Enables development of cross-cancer lymphocyte-assessment algorithms. Patient-level data division supports robust AI model validation, advancing generalized lymphocyte-detection tools.	https://lysto.grand-challenge.org/ (accessed on 18 July 2024)
PanNuke [84]	2020	Contains 200,000 nuclei categorized into five clinically significant classes, with high-resolution patches scanned at 20× or 40× magnification	Supports nuanced classification of different tissue types in breast cancer research	https://sites.google.com/view/panoptils (accessed on 18 July 2024)
NuClick [85]	2020	NuClick dataset comprises 871 images derived from 440 WSIs, strategically partitioned into 471 training, 99 validation, and 300 testing images. The dataset encompasses various cancer types, with careful consideration given to maintaining separation between patient samples across different sets.	Supports AI model development for cancer diagnostics with real-world applicability. Thoughtful data division prevents patient-level leakage, enhancing model reliability and clinical relevance.	https://github.com/navidstuv/NuClick (accessed on 18 July 2024)
IMPRESS [86]	2020	Large-scale linguistic dataset comprising over 25,000 sentence pairs. Utilizes a rich vocabulary of 3000+ lexical items with grammatical annotations. Generated using advanced natural language processing techniques, following established Natural language inference dataset formats.	Advances pragmatic inference research by providing a comprehensive benchmark for evaluating NLI models’ understanding of presuppositions and implicatures. Facilitates the development of more nuanced language understanding systems capable of grasping subtle linguistic phenomena.	https://github.com/facebookresearch/Imppres?tab=readme-ov-file (accessed on 18 July 2024)
BRACS [87]	2021	4539 ROIs from 547 H&E WSIs, meticulously categorized into different lesion types.	Facilitates detailed subtyping in breast cancer research.	https://www.bracs.icar.cnr.it/ (accessed on 18 July 2024)
SHIDC-BC-Ki-67 [38]	2021	2357 tru-cut biopsy images of invasive ductal carcinoma, collected from 2017 to 2020. Comprises 1656 training and 701 test samples, all expertly annotated for Ki-67 markers.	Addresses the scarcity of comprehensive Ki-67 marked datasets in breast cancer research. Facilitates development of deep learning models for accurate Ki-67 assessment, potentially enhancing diagnostic precision and treatment planning for invasive ductal carcinoma.	https://shiraz-hidc.com/ki-67-dataset/ (accessed on 18 July 2024)
MIDOG21 [87]	2021	280 breast cancer WSIs at 8000 × 8000 pixels, scanned by 4 different devices.	Addresses scanner variability in mitosis detection, promoting robust algorithm development across diverse imaging equipment.	https://midog2021.grand-challenge.org/ (accessed on 18 July 2024)
TIGER [88]	2022	Includes H&E-stained WSIs of Her2 positive and Triple Negative breast cancer. TIGER comprises cases from clinical routine and a phase 3 clinical trial. Includes annotations for lymphocytes, plasma cells, invasive tumors, and stroma.	First challenge for fully automated assessment of TILs in breast cancer. Aims to validate AI-based TIL scores for clinical use.	https://tiger.grand-challenge.org/ (accessed on 18 July 2024)
MIDOG22 [89]	2022	50 canine mast cell tumor cases, scanned by a single device type.	Expands mitosis detection to veterinary pathology, offering cross-species validation for detection algorithms.	https://midog.deepmicroscopy.org/download-dataset/ (accessed on 18 July 2024)
PanopTILs [65]	2023	Includes annotations for 814,886 nuclei from 151 patients, with WSIs scanned at 20× magnification.	Enhances understanding of the role of TILs in breast cancer detection.	https://sites.google.com/view/panoptils (accessed on 18 July 2024)
AI-TUMOR [73]	2024	2500 WSIs with pixel-level annotations for tumor regions, normal tissue, and surrounding stroma.	Focuses on reducing biases in AI models, ensuring better generalizability.	https://github.com/nickryd/ATLAS/blob/main/README.md (accessed on 18 July 2024)

**Table 5:** Main challenges in WSIs for comprehensive breast cancer assessment.

Challenge	Description	Impact on Breast Cancer Cell Detection	Source
Size and Complexity of WSIs	WSIs can be several gigabytes in size, containing millions of pixels that need to be processed.	Requires substantial computational resources, high-performance GPUs, and large memory capacities; storage and management of large datasets are logistically challenging.	[28,29,30]
Variability in Image Quality and Resolution	Inconsistencies in image quality and resolution across different scanners and datasets.	Leads to variations in training data quality, reducing model generalization and accuracy across different scanners.	[27,28,31,39,42,45,51,54,63,64]
Annotation Challenges	High-quality annotations are labor-intensive, time-consuming, and prone to variability.	Inconsistent annotations can introduce biases, affecting model robustness and generalizability.	[29,40,47,57,60,61]
Feature Extraction Difficulties	Complex and subtle patterns in breast cancer tissues are challenging to capture accurately.	Traditional methods often fall short, necessitating advanced deep learning techniques for accurate detection.	[29,30,32,33,34,35,36,37,38,40,41,43,44,45,46,47,48,49,50,51,52,53,54,55,56,57,58,59,60,62,63,65]

**Table 6:** The impact of WSIs on the accuracy and reliability of deep learning methods for comprehensive breast cancer assessment.

Factor	Description	Impact on Accuracy and Reliability
Resolution of WSIs	High-resolution images provide detailed information, crucial for detecting subtle differences between normal and cancerous tissues, especially in early-stage cancers.	Enhances detection accuracy but increases computational requirements.
Quality and Consistency	Variations in staining, lighting, and scanner types can introduce noise and artifacts, affecting model predictions.	Inconsistent quality can lead to inaccuracies; standardization techniques are vital.
Availability of Annotated WSIs	High-quality annotations by expert pathologists are essential for training accurate models. Obtaining such annotations is resource-intensive.	Poor annotations reduce model accuracy; improving annotation quality is key.
Diversity and Generalizability of Training	Models trained on diverse WSIs, covering a range of tissue types and patient demographics, are more likely to generalize well across different clinical settings.	Enhances generalization and reliability, but lack of standardization can hinder performance.

**Table 7:** Baseline models for WSI detection technologies and the strategies for optimizing and improving these models based on specific criteria.

Criteria	Baseline Models	Optimization and Improvement Strategies
Accuracy and Performance Metrics	CNN, GAN, U-Net, Transformer, MIL, MLP	Ensemble learning frameworks Multi-task learning paradigms Multimodal data fusion strategies Cross-institutional training Advanced convolutional modules Multiple attention mechanisms
Robustness and Generalizability	CNN, U-Net, Transformer	Cross-disease data integration and validation Semi-supervised learning paradigms
Interpretability and Explainability	CNN, MIL	HIF approach Saliency visualization techniques
Computational Efficiency	CNN, U-Net, YOLO	Precise localization of ROI Pre-training strategies
Annotation Quality and Requirements	CNN, U-Net	Weak supervision learning Segmentation map generation techniques

## Data Availability

Not applicable.

## Abbreviations

The following abbreviations are used in this manuscript:

PRISM	Preferred Reporting Items for Systematic Reviews and Meta-Analyses
WSI	Whole Slide Image
WSIs	Whole Slide Images
ER	Estrogen Receptor
PR	rogesterone Receptor
HER2	Human Epidermal Growth Factor Receptor 2
Ki-67	Ki-67 Antigen
TIL	Tumor-Infiltrating Lymphocyte
H&E	Hematoxylin and Eosin
IHC	Immunohistochemistry
TNBC	Triple-Negative Breast Cancer
LD	Lymphocyte Detection
BD	Biomarker Detection
SCQ	Systematic Quality Criteria
SLR	Systematic Literature Review
HPF	High-Power Field
CNN	Convolutional Neural Network
GAN	Generative Adversarial Network
MLP	Multilayer Perceptron
MIL	Multiple Instance Learning
YOLO	You Only Look Once
CAD	Computer-Aided Diagnosis
ROI	Region of Interest
HIF	Human-Interpretable Features
